# C-Type Lectin-Like Receptors As Emerging Orchestrators of Sterile Inflammation Represent Potential Therapeutic Targets

**DOI:** 10.3389/fimmu.2018.00227

**Published:** 2018-02-15

**Authors:** Elise Chiffoleau

**Affiliations:** ^1^Centre de Recherche en Transplantation et Immunologie UMR1064, INSERM, Université de Nantes, Nantes, France; ^2^Institut de Transplantation Urologie Néphrologie (ITUN), CHU Nantes, Nantes, France; ^3^IHU Cesti, Nantes, France; ^4^Labex Immunotherapy Graft Oncology (IGO), Nantes, France

**Keywords:** C-type lectin-like receptors, sterile inflammation, autoimmune diseases, tissue injury, cancer

## Abstract

Over the last decade, C-type lectin-like receptors (CTLRs), expressed mostly by myeloid cells, have gained increasing attention for their role in the fine tuning of both innate and adaptive immunity. Not only CTLRs recognize pathogen-derived ligands to protect against infection but also endogenous ligands such as self-carbohydrates, proteins, or lipids to control homeostasis and tissue injury. Interestingly, CTLRs act as antigen-uptake receptors *via* their carbohydrate-recognition domain for internalization and subsequent presentation to T-cells. Furthermore, CTLRs signal through a complex intracellular network leading to the secretion of a particular set of cytokines that differently polarizes downstream effector T-cell responses according to the ligand and pattern recognition receptor co-engagement. Thus, by orchestrating the balance between inflammatory and resolution pathways, CTLRs are now considered as driving players of sterile inflammation whose dysregulation leads to the development of various pathologies such as autoimmune diseases, allergy, or cancer. For examples, the macrophage-inducible C-type lectin (MINCLE), by sensing glycolipids released during cell-damage, promotes skin allergy and the pathogenesis of experimental autoimmune uveoretinitis. Besides, recent studies described that tumors use physiological process of the CTLRs’ dendritic cell-associated C-type lectin-1 (DECTIN-1) and MINCLE to locally suppress myeloid cell activation and promote immune evasion. Therefore, we aim here to overview the current knowledge of the pivotal role of CTLRs in sterile inflammation with special attention given to the “Dectin-1” and “Dectin-2” families. Moreover, we will discuss the potential of these receptors as promising therapeutic targets to treat a wide range of acute and chronic diseases.

## Introduction

**Figure 1:**
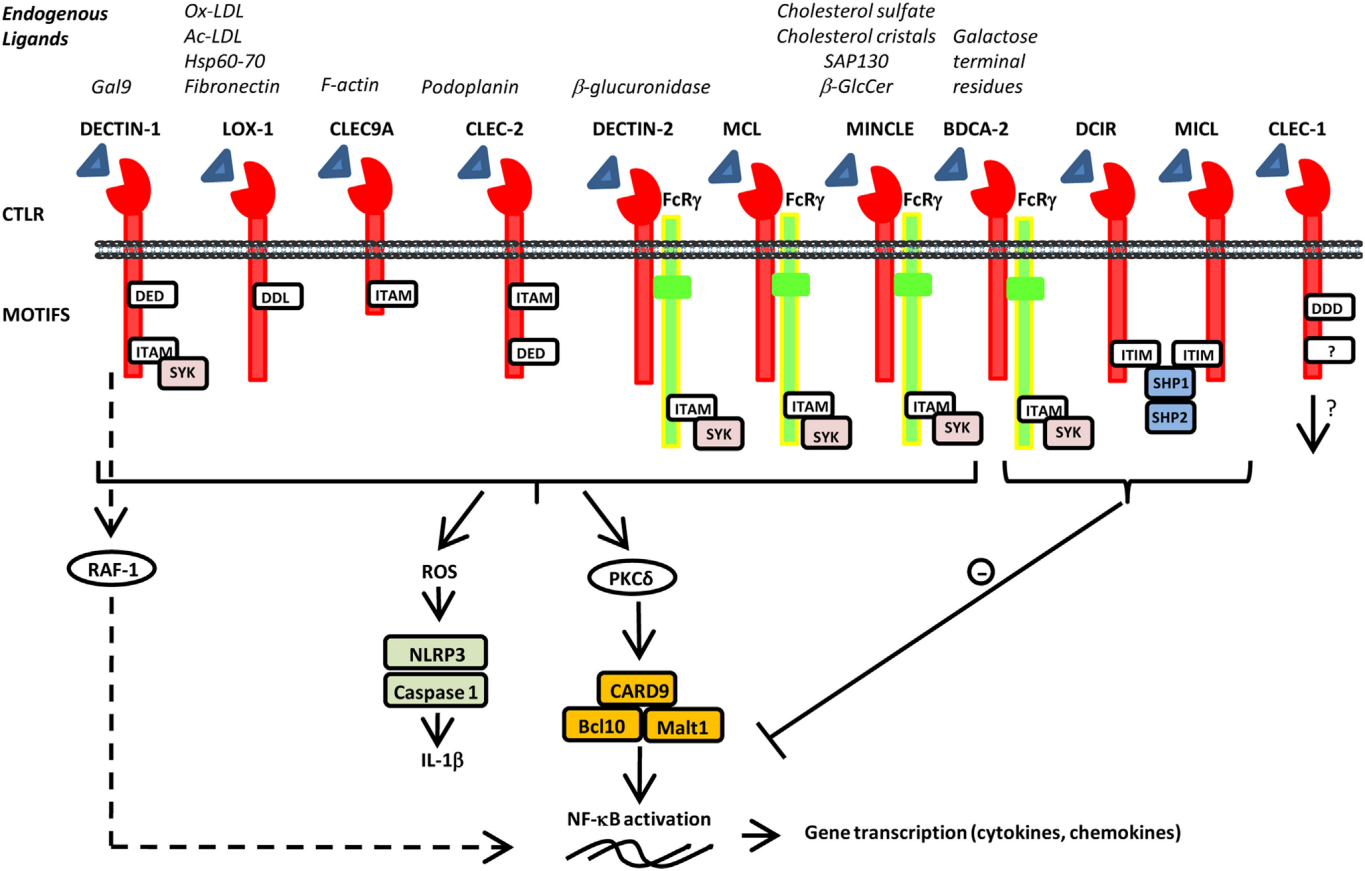
Schematic representation of various C-type lectin-like receptors (CTLRs) and selected endogenous ligands and signals. CTLRs are composed of an extracellular C-type lectin-like domain able to recognize various endogenous ligands and signal directly, through integral motifs in their cytoplasmic tails or indirectly through association with FcRγ. They can also contain a tri-acidic domain DED or DDD important for phagocytosis. Activation of immune-receptor tyrosine-based activation motif (ITAM) leads to the recruitment and activation of SYK family kinases. Subsequent activation of the CARD9–Bcl10–Malt1 complex through PKδ induces NF-κB activation and gene transcription of various cytokine and chemokines. Furthermore, SYK induces reactive oxygen species production and inflammasome activation *via* NLRP3 and Caspase 1 leading to IL-1β production. Alternative pathway of signalization independently of SYK has been reported for dendritic cell-associated C-type lectin-1 (DECTIN-1) *via* RAF-1 to finely regulate NF-κB activation. By contrast, activation of immune-receptor tyrosine-based inhibition motif (ITIM) induces the recruitment and activation of protein tyrosine phosphatases such as SHP-1 and SHP-2 and the dephosphorylation of motifs to inhibit cellular activation mediated by other immunoreceptors.

C-type lectin receptors (CLRs) are a large family of transmembrane and soluble receptors that contain one or more carbohydrate-recognition domain able to recognize a wide variety of glycans on pathogens or on self-proteins. The hallmark of classical CLRs is the dependence on Ca^2+^ for glycan recognition. However, many other CLRs lack the coordinated Ca^2+^ ions and are therefore referred as C-type lectin-like molecules. These C-type lectin-like receptors (CTLRs) are still able to recognize carbohydrates but independently of Ca^2+^ but also recognize more diverse ligands such as lipids and proteins (1). Of particular interest for their role in coupling both innate and adaptive immunity, are the CTLR genes of the “*Dectin-1*” and “*Dectin-2*” families localized on the telomeric region of the natural killer cluster of genes (2, 3). These two groups of CTLRs are expressed mostly by cells of myeloid lineage such as monocytes, macrophages, dendritic cells (DCs), and neutrophils. CTLRs not only serve as antigen-uptake receptors for internalization and presentation to T cells but also trigger multiple signaling pathways leading to NF-κB, type I interferon (IFN), and/or inflammasome activation (1–4). This leads, in turn, to the production of pro- or anti-inflammatory cytokines and chemokines, subsequently fine tuning adaptive immune responses. CTLRs can signal either directly, through integral signaling domains, or indirectly, by associating with adaptor molecules. As illustrated in Figure 1, activation of immune-receptor tyrosine-based activation motif (ITAM) directly or *via* adaptor proteins such as FcγR, leads to the recruitment of SYK family kinases and the formation of the Card9/Bcl10/Malt1 complex that downstream activates NF-κB pathway and various cellular responses. By contrast, activation of immune-receptor tyrosine-based inhibition motif (ITIM) induces the recruitment and activation of protein tyrosine phosphatases such as SHP-1 and SHP-2 and the dephosphorylation of motifs (1). Consequently, ITIM signaling can inhibit cellular activation mediated by other immunoreceptors to tightly regulate immune response. Such checkpoints allow to prevent uncontrolled immune responses that may lead to harmful, or even fatal, consequences. In addition, some CTLRs were also reported to signal *via* SYK-independent pathway through the serine/threonine kinase RAF-1 to drive particular Th differentiation (5). Besides, by integrating simultaneous signals from other pattern recognition receptors (PRRs), CTLRs can exert synergistic or antagonistic response to achieve appropriate biological responses (6). This cross talk is regulated by the level and localization of their expression, by their interaction and by their collaborative or conflicting signaling (6, 7). To date, CTLRs “Dectin” families were best known for their involvement in host defense as referred in these excellent reviews (1–4, 8, 9). However, over recent years, these receptors have gained growing interest for their ability to respond also to a wide variety of endogenous ligands (Figure 1). Identification of self-glycans, lipids, or proteins expressed or released by modified or damaged cells reinforced the hypothesis for their implication in sterile inflammation whose dysregulation foster the development of wide range of diseases (10). In this mini review, we aim to focus on some of the CTLRs of the “Dendritic cell-associated C-type lectin (Dectin)” families, discussing the recent discoveries on their implication in the control of tissue injury, autoimmune diseases, or tumorigenesis. In addition, we will underscore their therapeutic potential and impact on human health.

## “(DECTIN-1)” Family

### DECTIN-1 (Alias CLEC7A, CLECSF12, CANDF4, CD369, BGR)

The CTLR, DECTIN-1 has been reported to be enhanced by pro-inflammatory conditions (11, 12) and to be a potent inducer of Th1 and/or Th17 responses in response to pathogens (2). Thereby, pathogenic ligands of DECTIN-1 are currently used to bolster immune responses notably in cancer. For example, administration of β glucans was shown to inhibit tumor growth in murine carcinoma models (13–15), in human melanoma, neuroblastoma, mastocytosis, and lymphoma xenograft models (16, 17) and in ovarian (18, 19), breast (20), lung (14, 21–23), and gastric cancer (19, 24). Mechanistically, β glucans were shown to convert immunosuppressive macrophages into an M1-like antitumoral phenotype (25), to promote NK (26) and CD8^+^ T cell cytotoxicity (27) as well as a decrease in myeloid-derived suppressor cells and regulatory T cells (13, 28). Interestingly, Zhao et al. recently reported that β glucans upregulate particularly the expression of TNFSF15 and OX40L in DCs in mice, thus promoting efficient Th9 priming and potent anti-melanoma response following vaccination (29). On the contrary, some investigations have described an inhibitory function of DECTIN-1 in sterile inflammation notably during hepatic fibrosis and hepatocellular carcinoma (30). Authors showed that DECTIN-1 inhibit TLR4 signaling and downstream inflammation such as TNFα, IL-6, and chemokines secretion (30). Moreover, DECTIN-1 was reported to be associated with mechanisms of peritumoral immune tolerance by programming suppressive macrophages in pancreatic ductal adenocarcinoma (31). Strikingly, they showed that blockade of DECTIN-1 or its endogenous ligand Galectin-9, both strongly expressed on infiltrating myeloid cells and tumor, delayed tumor progression and extended mice survival. A similar tolerogenic signal of DECTIN-1 has been shown in myeloid cells in response to mucus in the intestine through interaction with Galectin-3 (32). In addition, DECTIN-1-deficient mice were described to exacerbate inflammation in a model of colitis suggesting an important role of DECTIN-1 in gut homeostasis (33). Therefore, DECTIN-1 seems to act as double-edged swords on the regulation of inflammation. Such discrepancy may depend of the type of the response, the nature and the property of the ligands, and of the complex signal network that integrates diverse engaged PRRs.

### LOX-1 (Alias OLR-1, CLEC8A)

The CTLR lectin-like oxidized low-density lipoprotein receptor-1 (LOX-1) is particularly expressed by endothelial cells and platelets and is upregulated during inflammatory and pathological conditions (34–36). By recognizing oxidized and acetylated low-density lipoproteins, LOX-1 is largely described to play critical functions in vascular diseases, including atherosclerosis (37). However, recent investigations have revealed that LOX-1 is also expressed by human macrophages (38) and DCs (39), and its triggering increases secretion of IL-6 to potentiate B-cell class-switch (39). Moreover, LOX-1 enhances CCR10, APRIL, and BAFF secretion for plasma cell differentiation and migration to mucosal site. In line with these findings, targeting influenza histocompatibility antigen-1 to LOX-1 elicits antigen-specific protective antibody response to virus in macaques suggesting a good candidate for vaccine development (39). Besides, several studies have reported a high expression of LOX-1 in various tumors including gastric (40), colorectal (41), and prostate (42) cancers, which correlates with a poor prognosis in patients (40). Functionally, LOX-1 was shown to promote tumor angiogenesis (42), metastasis (43), and the migration and invasion of gastric cancer cells by notably driving epithelial–mesenchymal transition (40). Interestingly, LOX-1 was recently identified to be particularly expressed by potent polymorphonuclear myeloid-derived suppressor cells from blood and tumor of patients with non-small cell lung or head neck cancer and to be associated with worse survival (44). In fact, LOX-1 expression seems to be upregulated following endoplasmic reticulum stress that occurs during hypoxia or nutrient deprivation inside tumors. These data render this marker an attractive therapeutic target as well as a diagnostic tool for cancer screening (41).

### CLEC-1 (Alias CLEC1, CLEC1A)

Although the C-type lectin-like receptor-1 (CLEC-1) was identified a long time ago (45, 46), the downstream signaling and ligand(s) remain uncharacterized (8, 47). We and others described CLEC-1 expression in human and rodent by myeloid cells such as monocytes, DC, and macrophages but also by endothelial cells (8, 9, 46, 48). CLEC-1 expression is decreased by pro-inflammatory stimuli and is enhanced by TGFβ (8, 9, 48). Interestingly, CLEC-1 was found to be expressed mostly intracellular particularly in human endothelial cells and neutrophils (8, 9), suggesting the requirement of particular conditions for cell-surface expression or for recycling from intracellular pools (7). Alternatively, CLEC-1 may play a role in intracellular organelles. Using CLEC-1-deficient rodents, we showed that disruption of CLEC-1 signaling enhances *Il12p40* subunit expression in DCs and accordingly exacerbates downstream CD4^+^ Th1 and Th17 responses following *in vivo* immunization with exogeneous antigens (9, 48).

### CLEC-2 (Alias CLEC2, CLEC2B, CLEC1B)

C-type lectin-like receptor 2 (CLEC-2) is found on platelets and DCs and is largely described for its interaction with its endogeneous ligand podoplanin expressed by lymphatic endothelial cells, myeloid cells, and fibroblast reticular cells (2). The CLEC-2/Podoplanin axis was shown to be critical in platelet activation (49), lymph node microarchitecture (50, 51), reticular network (52), and vascular integrity. Besides, this interaction promotes tumor cell-induced platelet aggregation, tumor growth, and metastasis (53–56) in various types of cancer including brain, lung, and larynx (57–60). Furthermore, CLEC-2 is enhanced by inflammation, promotes DC migration (61) and together with LPS enhances the production of the anti-inflammatory cytokine IL-10 suggesting also a role in the resolution of inflammation (62).

### MICL (Alias CLEC12A, DCAL-2, CLL1, CLL-1, KLRL1)

Myeloid inhibitory C-type lectin-like receptor (MICL) is expressed predominantly by granulocytes and monocytes, and its expression is downregulated by pro-inflammatory stimuli (63–65). MICL recruits inhibitory phosphatases and again seems to differently shape T-cell responses according to the cross talk with simultaneous PRR signals. Chen and colleges demonstrated that co-engagement of MICL with TLR4 suppress IL-12 expression in human DCs and downstream Th1 polarization whereas co-engagement with CD40 does the opposite (66). Interestingly, putative endogenous ligands of MICL were identified on various mouse tissues in steady-state conditions, proposing a role for MICL in the control of homeostasis and self-tolerance (65). Corroborating this notion, an inhibitory function for MICL has been put in light in an *in vivo* model of induced rheumatoid arthritis (67). In an original way, MICL was proposed to modulate myeloid cell activation threshold by acting as an autoantigen during arthritis development (67).

### CLEC9A (Alias DNGR1, DNGR-1, CD370)

CLEC9A is selectively expressed on the mouse subsets of CD8α^+^ and CD103^+^ DCs, and on their human BDCA3^+^ DCs counterparts (68). CLEC9A expression is lost further TLR-induced maturation. Importantly, CLEC9A by recognizing F-actin released by necrotic cells is capable of internalizing bound dead cell-associated antigens for cross-presentation to CD8^+^ T cells (69–71). Thereby, CLEC9A has been demonstrated to be a powerful target for peptide vaccination to boost antitumor immunity (72, 73). Interestingly, it has recently been shown that necrotic debris that accumulated during atherosclerosis development, trigger through CLEC9A, the downregulation of the anti-inflammatory cytokine IL-10 and the disease progression (74).

## “DECTIN-2” Family

### DCIR (Alias CLEC4A, CLECSF6, CD367, LLIR)

DC immunoreceptor (DCIR) is expressed on monocytes, neutrophils, DC, and plasmacytoid DCs, and its expression is decreased by pro-inflammatory stimuli (75). The human genome encodes only a single DCIR gene, whereas the mouse genome presents four DCIR-like genes (DCIR1–4) (76). DCIR *via* its canonical ITIM domain is largely recognized to exert inhibitory cross talk with other PRRs to maintain immune homeostasis and prevent excessive detrimental inflammation and immunopathogenesis (77–79). DCIR inhibits TLR8-induced IL-12 and TNFα production in human moDCs following cross-linking with monoclonal antibody (79). Furthermore, DCIR1 KO mice develop a late spontaneous autoimmune disease associated with elevated levels of autoantibodies, are more susceptible to collagen-induced arthritis, and aggravated experimental autoimmune encephalomyelitis (80, 81). These effects were described to be mediated at least by unrestrained growth of DC population in these mice. However, in support for a role of DCIR in tempering DC activation, a recent study demonstrated that DCIR2 selectively expressed by mouse CD8α^−^ DCs, strongly moderates pro-inflammatory and downstream T-cell responses (82). *In vivo*, DCIR2-deficient mice are more susceptible to endotoxic shock and aggravate experimental autoimmune encephalomyelitis development by increasing both Th1 and Th17 differentiation. Authors demonstrated that putative endogeneous ligands of DCIR are expressed also on cell surface of DCs. In line with these data, DCIR2 was described in DCs to sustain STAT-1 type I IFN signaling leading to a reduction of IL-12p70 production and Th1 differentiation in response to endogeneous ligand(s) released during cell culture (83). Therefore, by regulating also the IFN responses, DCIR may be a critical player in the control of a number of inflammatory diseases. Interestingly, DCIR was also reported to bind to commensal intestinal microbes (84). However, DCIR-deficient mice only exhibit a slightly increased severity of colitis in a dextran sulfate sodium model (84).

### DECTIN-2 (Alias CLEC6A, CLEC4N, CLECSF10)

Several studies suggest a role for DC-associated C-type lectin-2 (DECTIN-2) in the inhibition of sterile inflammation. DECTIN-2 is enhanced in pro-inflammatory conditions and was shown notably to bind to a putative ligand on regulatory CD4^+^CD25^+^ T cells to mediate ultraviolet radiation-induced tolerance (85, 86). In addition, DECTIN-2 recognizes glycan mannose on the lysosomal enzyme β-glucuronidase, known to moderate arthritis pathogenesis by preventing accumulation of pro-inflammatory glycosaminoglycans within inflamed joint tissue (87–90). Thus, β-glucuronidase released by dead myeloid cells following tissue damage may act *via* DECTIN-2 as an inhibitory loop in DCs to temper inflammation (91). Besides, polymorphism of this enzyme was reported to be associated with mucopolysaccharidoses characterized by a pro-inflammatory response (92). In addition, a role for DECTIN-2 in suppression of liver metastasis has been highlighted by its ability to phagocytose cancer cells *via* CD11b F4/80 Kupffer cells during extravasation step (93).

### BDCA-2 (Alias CLEC4C, BDCA2, CD303, CLECSF11, CLECSF7)

Interestingly, blood DC antigen-2 (BDCA-2) is the most specific marker for human plasmacytoid DC but intriguingly is not expressed in mice (94). Expression of BDCA-2 is downregulated following maturation (95). Surprisingly, unlike many other ITAM-coupled receptors, signaling through BDCA-2 inhibits activation of the NF-κB pathway and the production of type I IFNs and cytokines in response to TLR9 ligands or following recognition of galactose terminal residues notably expressed on tumor cells (94, 96, 97). BDCA-2 engagement was also shown to block TRAIL-mediated cytotoxic activity (98). In an interestingly way, BDCA-2 was suggested to function as an Fc receptor by binding glycans on immunoglobulins G (99) and thus, dampens down inflammation in response to rising levels of serum immunoglobulins G.

### MINCLE (Alias CLEC4E, CLECSF9)

**Figure 2:**
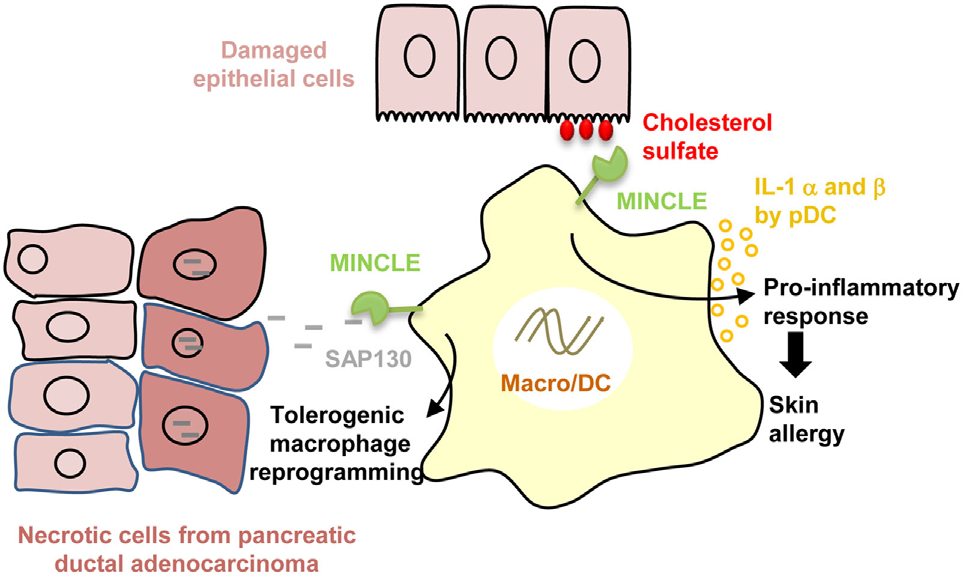
Dual role of macrophage-inducible C-type lectin (MINCLE) in disease pathogenesis. Recognition of cholesterol sulfate by MINCLE whose expression is increased in plasmacytoid dendritic cells (DC) following skin damage, induces IL-1 α and β secretion, and promotes skin allergy and allergic contact dermatitis. By contrast, MINCLE recognition of spliceosome-associated protein 130 (SAP130) released by necrotic cancer cells leads to tolerogenic tumor-infiltrating macrophage reprogramming in pancreatic ductal adenocarcinoma.

Macrophage-inducible C-type lectin (MINCLE) is an ITAM-coupled CTLR that forms a heterodimer with the macrophage C-type lectin (MCL). MINCLE expression is enhanced after exposure to pro-inflammatory stimuli or cellular stresses and is translocated to the cell surface *via* interaction with the stalk region of MCL (100–104). MINCLE was shown to activate in DCs both NF-κB and inflammasome to greatly enhance IL-1β expression in synergy with TLR7/8 (R848) or following *in vivo* immunization with Freund adjuvant (105, 106). MINCLE senses self-damage by recognizing “unfamiliar” glycolipids that are not present in the extracellular milieu under normal, healthy conditions. For example, MINCLE was reported to bind crystalline cholesterol present in atheriosclerotic plaques that are associated with inflammation and macrophage infiltrates (107). Likewise, as depicted in Figure 2, MINCLE is enhanced on plasmacytoid DCs following skin damage and by recognizing cholesterol sulfate, induces IL-1 α and β secretion, and promotes skin allergy and allergic contact dermatitis (108). Moreover, MINCLE was also reported to bind to the ubiquitous intracellular metabolite β-glucosylceramide released by damaged cells to promote production of pro-inflammatory cytokines by myeloid cells (109). In an opposite way, recent investigations have revealed that MINCLE rather than purely inducing pro-inflammatory responses can also promote the expression of the anti-inflammatory cytokines IL-10 (110). In addition, MINCLE was reported to counter regulate pro-inflammatory signaling pathways mediated by DECTIN-1 to temper IL12p35 production (6, 111). Therefore, MINCLE seems to also exert opposite role on immune responses depending of the ligands and PRR interference. This dual effect is illustrated by the recognition by MINCLE of the spliceosome-associated protein 130 (SAP130), a component of small nuclear ribonucleoprotein released during non homeostatic cell death. On one hand, MINCLE/SAP130 axis was shown to be involved in the pathogenesis of inflammation during tissue damages (112) or ischemia/reperfusion (113, 114) and to contribute to the development of experimental autoimmune uveoretinitis (115). This pro-inflammatory side of MINCLE is supported by a high expression of MINCLE in patients with rheumatoid arthritis (116) and by the link to arthritis of the rat chromosome 4q42 encoding *Mincle* (117). On the other hand, in the context of cancer, MINCLE/SAP130 axis was reported to be pro-tumorigenic in mouse and human pancreatic ductal adenocarcinoma (118). Both MINCLE and SAP130 released by programmed necrosis are highly expressed in mouse and human carcinoma and as depicted in Figure 2, this interaction leads to an immunosuppressive reprogramming of infiltrating myeloid cells (118). Future research is required to provide insight as to how MINCLE needs to integrate with other PRR signals to differently define the type of immune response.

## Therapeutic Potential of CTLRs

Therefore, by their capacity to present antigen and ensure the balance between cellular activation and suppression, CTLRs have emerged as challenging pharmacological targets to treat a wide variety of diseases governed by sterile inflammation including cancers, autoimmune diseases or allergy (1, 119). Ligands such as carbohydrate structures, antibodies, or mimetic peptides could be therapeutically exploited as agonists or antagonists of CTLR signaling. As previously mentioned, the DECTIN-1 agonist β-glucans is used to elicit of potent antitumor immune responses in various types of cancer (14, 16–24). Furthermore, CTLRs such as DEC-205 (120, 121) or CLEC9A (71) have been exploited for the *in vivo* delivery target of vaccine antigens in cancer (122). In addition, synthetic ligands of MINCLE were generated to specifically enhance immune response (102). Besides, several specific antibodies generated against cancer-specific highly glycosylated podoplanin were shown to efficiently block the CLEC-2/Podoplanin interaction, subsequent platelet aggregation and tumor metastasis (123–128). Importantly, a particular antibody that reacts with podoplanin-expressing cancer cells but not with the one from normal cells has been successfully generated and will be useful for molecular targeting therapy against podoplanin-expressing cancer cells only (126). However, since CTLRs have overlapping ligands that induce distinct and even contrasting immune responses, antibodies targeting specific CTLRs could be more appropriate. Also, inhibitors such as recombinant peptide spanning the CTLR binding region and modulating the receptor–ligand interaction could be considered. Only a few drug-like molecules have been developed for the CTLR family (129) but studies indicate high *in silico* druggability scores as well as high experimental hit rates from peptide fragment screenings (130, 131).

To conclude, CTLR modulation seems to represent promising strategy for disease management although attempts at identifying endogenous ligands as well as efforts to elucidate their role in sterile inflammation are still warrant.

## Author Contributions

The author confirms being the sole contributor of this work and approved it for publication.

## Conflict of Interest Statement

The author declares that the research was conducted in the absence of any commercial or financial relationships that could be construed as a potential conflict of interest.
